# Association between XmnI Polymorphism and HbF Level in Sickle Cell Disease Patients from Chhattisgarh

**Published:** 2012-03

**Authors:** Sanjana Bhagat, Pradeep Kumar Patra, Amar Singh Thakur

**Affiliations:** 1 *Department of Biochemistry, Pt. J. N. M. Medical college, Raipur, Chhattisgarh, India;*; 2 *Department of Biochemistry, Govt. Medical college, Jagdalpur, Chhattisgarh, India*

**Keywords:** HbF level, sickle cell anemia, globin polymorphism

## Abstract

The γ^G^-158 (C→T) polymorphism plays important function in the disease severity of sickle cell anemia. The XmnI restriction site at -158 position of the γ^G^-gene is associated with increased expression of the γ^G^-globin gene and higher production of HbF. This study aims to determine the frequency of the different genotypes of the γ^G^ Xmn I polymorphism in sickle cell anemia and sickle cell trait patients in Chhattisgarh and its association with high HbF level. The Xmn1 polymorphic site was determined by PCR-RFLP procedure. XmnI polymorphism were studied in 100 sickle cell patients (SS), 50 sickle cell trait (AS) and 50 controls individuals (AA). The presence of XmnI (+/+) site in SS and AS patients associated with the increase of HbF (*P*<0.0001) synthesis. we also find that presence of one XmnI (+/-) site in SS patients compared with XmnI-/- site had not shows difference in HbF level. Polymorphic association is found between presence and absence of XmnI site with HbF level, in AS and AA individuals.

## INTRODUCTION

Sickle cell anemia (SCA) is a common form of hereditary disease in Chhattisgarh with a highly variable phenotype characterized by painful episode, enlarged spleen, serious frequent infections, vaso-occlusive crisis and acute clinical events. Clinical severity of sickle cell disease is extremely variable, and the reasons for this heterogeneity are not fully understood. Although the risk factors underlying these complications are not well characterized, higher expression of fetal haemoglobin (HbF) in adulthood ameliorates morbidity and mortality in sickle cell disease (1-5). Thus, inter individuals variations in HbF levels is likely one of the main modifiers that contribute to the clinical heterogeneity observed in sickle cell disease patients.

Variable increases in HbF levels have been noted in individuals with sickle cell anemia, which are caused by mutation affecting the HBB gene and inherited as Mendelian recessives. Individuals with SCA have HbF levels ranging from 1-30% (6). An indication that additional variation at the β globin cluster is responsible for some of this variability came from the discovery of the ‘sickle β haplotype’, and that the β^S^ gene on certain β^S^ haplotype are associated with higher HbF levels and a milder disease (7, 8).

The γ^G^-158 (C→T) polymorphism (-158 Xmn I γ^G^ globin polymorphism) has been shown to be associated with the increased production of HbF and can strongly influences this heterogeneity of sickle cell anemia (9-11). The condition of the (-158 Xmn I γ^G^ globin polymorphism has not been reported in sickle cell anemia patients from Chhattisgah. The present study was to investigate the frequency of the -158 XmnI γ^G^ globin polymorphism and its association with high HbF level in sickle cell disease patients of the Chhattisgarh.

## METHODS

Sickle cell anemia (SS) [100], sickle cell trait (AS) [50] patients were taken into consideration and [50] control individuals (AA) for comparison of results. Patients with other associated disease, belonging to same family, patients under Hydroxyurea treatment and blood transfusion cases were excluded. Primary screening (Solubility test and Cellulose Acetate Electrophoresis) was performed prior to selection of patients). Control individuals have not represented any frequent clinical symptoms. Mean age of SS was 20.3 years, AS was 26.12 years and control AA was 24.52 years. Because of the influence of age on the HbF level, patients younger than five years were excluded from the study.

HbF levels was determined by HPLC (*VARIANT^TM^ β – thalassemia Short Program* (*Bio-Rad*)).

The 5 ml blood was collected after obtaining proper consent from all individuals. Genomic DNA was extracted from white blood cells by phenol-chloroform. The sickle cell mutation was confirmed by amplifying the 5’ region of the β-globin gene followed by restriction digestion with DdeI. A 650-bp fragment 5’ to the γ^G^ gene was amplified using the primer 5’- aac tgt tgc ttt ata gga ttt t-3’ and 5’- agg agc tta ttg ata acc tca gac-3’. The amplification condition were initial denaturation 94°C for 5 min followed by 30 cycles of 94°C for 1 min, 55°C for 1 min, 72°C 1 min, with a final extension of 5 min at 72°C. The PCR product was digested with three unit of XmnI restriction enzyme and separated by electrophoresis on 3% agarose gel.

Two- tailed Student’s t test was used for statistical analysis. Result were expressed as mean ± SD. *P*<0.05 was considered as statistically significant.

## RESULT

The prevalence of XmnI polymorphic site in patients of sickle cell anemia, trait and control is depicted in Table 1. Sixteen percent of patients with SS had two XmnI sites, fifty-five (55%) of the patients had one XmnI site. Most (40%) individuals with AS trait, had one XmnI site. XmnI (-/-) was the predominant state (76%) in control individuals. The frequency of polymorphism in sickle cell patients was significantly higher than those in sickle cell traits and normal controls.

**Table 1:** The HbF percentage and XmnI status in SS, AS and AA group

XmnI	SS (n=100)	HbF (Mean ± SD)	AS (n=50)	HbF (Mean ± SD)	AA (n=50)	HbF (Mean ± SD)

+/+	16 (16%)	81.97 ± 3.30	4 (8%)	17.45 ± 0.58	2 (4%)	0.95 ± 0.63
+/-	55 (55%)	21.84 ± 13.83	20 (40%)	0.8 ± 0.57	10 (20%)	0.37 ± 0.22
-/-	29 (29%)	20.28 ± 10.86	26 (52%)	0.04 ± 0.11	38 (76%)	0.02 ± 0.08

Significantly different comparing XmnI +/+ with XmnI +/- (*P*<0.0001) or comparing XmnI +/+ with -/- (*P*<0.0001) in sickle cell patients(SS); Significantly different comparing XmnI +/+ with XmnI +/- (*P*<0.0001) or comparing XmnI +/- with -/- (*P*<0.0001) in sickle cell trait patients(AS); Significantly different comparing XmnI +/- with XmnI -/- (*P*<0.0001) or comparing XmnI +/+ with -/- (*P*<0.05) in normal control (AA).

**Figure 1:**
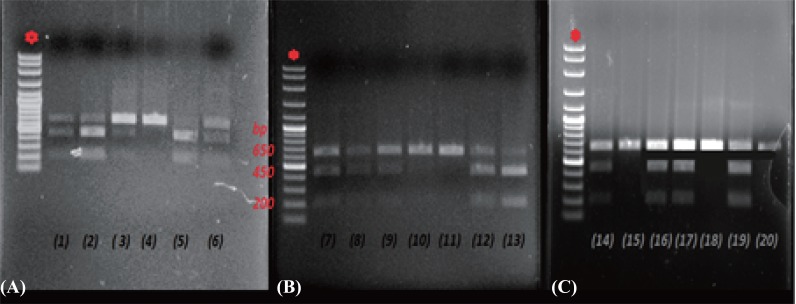
An agarose gel (A, B, C) shows electrophoresis pattern of RFLP product of XmnI site. First lane* of all three gel shows the molecular weight marker with 100 bp DNA ladder. Lane 4,10,11,15,18 and 20 shows RFLP product with XmnI (-/-) site. Lane 5 shows XmnI (+/+) site. Lane 1,2,6,7,8,9,12,13,14,16,17 product with XmnI (+/-) site.

Fig. 1 shows the XmnI polymorphic restriction sites studied in the 3% agarose gel. In the presence of T allele, two fragments of 450 and 200-bp were produced. The presence of the normal allele C loses cleavage site for XmnI and thus an intact 650-bp fragment were produced.

The association of XmnI polymorphic site and the percentage of HbF in all patients and control are given in the Table 1. In sickle cell patients, the HbF level was significantly higher in those who had two XmnI sites (*P*<0.0001) as compared to those with had only one XmnI site and with had absent of site (*P*<0.0001). In patients with SS who had one XmnI site and XmnI-/- site not had difference in HbF levels. In AS patients presence of two XmnI +/+ site compared with one XmnI site (*P*<0.0001) and one XmnI site compared with XmnI-/- site (*P*<0.0001) had significant higher level of HbF.

In present study, HbF level among normal individuals (AA), shown significant difference (*P*<0.05) between presence of XmnI +/+ and absence of XmnI-/- site. In patients with SS, the HbF level was higher in those who had one or two XmnI sites as compared to those with the site absent. In patients with Sickle cell trait AS, and AA, only the presence of one and two XmnI site (+/+) compared to the absence of the site (-/-) was associated with a significant increase in the HbF level.

## DISCUSSION

In the present study shows that a DNA polymorphism of the XmnI site at -158 C→T in the γ^G^ promoter was found to be associated with higher expression of HbF in sickle cell and sickle cell trait patients. Subsequent independent studies confirmed the association between the XmnI-HBG2 T allele and increased HbF, as well as milder disease among individuals with sickle cell anemia and β thalassemia from different population groups (7, 12-14).

Statistical analyses have identified no evidence for dominance at the locus, suggesting an addtitive effect of the γ^G^-158 (C→T) polymorphism. The finding of the present study shows that fifty-five patients with SS had one XmnI (+/-) site, and absent of site XmnI (-/-) (Table 1) not had difference in HbF levels was observed. The same pattern was observed for sickle cell trait, and the HbF level was highest in XmnI (+/+), moderate in XmnI (+/-) and the lowest in XmnI (-/-). In agreement with our results, Labie *et al*. (14) found a high gamma globin expression in β-thalassemia associated with haplotype II lacking the XmnI site and suggested the possible involvement of several other mutation in the gamma gene.

Presence of the ‘T’ allele does not always dictate the presence of a high HbF phenotype, and high HbF has been associated with β haplotype that do not include this allele (7, 15, 16). Recently, other genetic association studies shown that several single nucleotide polymorphisms, associated with variation in the expression of HbF in sickle cell disease (17). These genetic studies have identified three known major HbF quantitative trait loci (QTLs) (Xmn1-HBG2, HBS1L-MYB intergenic region on chromosome 6q23, and BCL11A on chromosome 2p16) that account for 20–50% of the common variation in HbF levels in patients with SCA and β thalassemia, and in healthy adults (18-20).

The XmnI polymorphism is known to influence the γ^G^ gene expression in sickle cell anemia and to increased HbF concentrations in particular when they are under conditions of erythropoietic stress. In sickle cell disease and sickle cell trait patients of Chhattisgarh, the presence of this polymorphism is associated with high HbF level. This is the first report of the frequency of the -158 XmnI γ^G^-globin polymorphism in patients with SS and AS in Chhattisgarh. In conclusion, our study on γ^G^-158 (C→T) polymorphism in sickle cell patients is associated with elevated level of HbF. Additionally, in our studies on Chhattisgarh, we did not observe any close link between the Xmn I polymorphic site and HbF levels. A wide range of HbF levels was obtained both in the presence and absence of this site.
